# Comparison of RetCam and Smartphone-Based Photography for Retinopathy of Prematurity Screening

**DOI:** 10.3390/diagnostics12040945

**Published:** 2022-04-10

**Authors:** Jui-Yen Lin, Eugene Yu-Chuan Kang, Alay S. Banker, Kuan-Jen Chen, Yih-Shiou Hwang, Chi-Chun Lai, Jhen-Ling Huang, Wei-Chi Wu

**Affiliations:** 1 Department of Ophthalmology, Chang Gung Memorial Hospital, Linkou Medical Center, Taoyuan 333, Taiwan; raylight1989@cgmh.org.tw (J.-Y.L.); yckang0321@gmail.com (E.Y.-C.K.); cgr999@cgmh.org.tw (K.-J.C.); yihshiou.hwang@gmail.com (Y.-S.H.); chichun.lai@gmail.com (C.-C.L.); 2 College of Medicine, Chang Gung University, Taoyuan 333, Taiwan; 3 Banker’s Retina Clinic and Laser Centre, Navrangpura, Ahmedabad 380009, India; bankersretinaclinic@gmail.com; 4 Center for Big Data Analytics and Statistics, Chang Gung Memorial Hospital, Taoyuan 333, Taiwan; cratwoy0309@gmail.com

**Keywords:** imaging systems, smartphone-based screen, telescreening, retinopathy of prematurity

## Abstract

This study aimed to compare the clinical performance between a smartphone-based fundus photography device and a contact imaging device for retinopathy of prematurity (ROP) screening. All patients were first examined with binocular indirect ophthalmoscopy (BIO), which served as the reference standard. The patients were then assessed by two devices. Imaging quality, ability to judge the zone and stage of ROP, agreement with the BIO results, vital signs, and pain scores were compared between these two devices. In total, 142 eyes of 71 infants were included. For the smartphone-based fundus photography, image quality was graded excellent or acceptable in 91.4% of examinations, although it was still significantly inferior to that of the contact imaging device (*p* < 0.001). The smartphone-based fundus photography images had moderate agreement with the BIO results regarding the presence or absence of plus disease (Cohen’s κ = 0.619), but evaluating the zone (*p* < 0.001) and stage (*p* < 0.001) of ROP was difficult. Systemic parameters, except for heart rate, were similar between the two imaging devices (all *p* > 0.05). In conclusion, although the smartphone-based fundus photography showed moderate agreement for determining the presence or absence of plus disease, it failed to identify the zone and stage of ROP.

## 1. Introduction

Retinopathy of prematurity (ROP) is a retinal vascular disorder related to prematurity and is one of the leading causes of blindness in the pediatric population. Because of the sequential nature of ROP progression, multiple studies have emphasized the need for a reliable screening protocol to ensure timely recognition of treatment-requiring ROP so that the appropriate treatment can be given, thus reducing the rate of blindness [1,2,3]. Although the importance of timely ROP screening is well recognized, few ophthalmologists have the proper training to meet the growing demand for ROP evaluations.

A potential solution to address this situation is to develop a telemedicine system that uses retinal imaging to detect treatment-requiring ROP. RetCam (RetCam 3, Natus Medical Inc., Pleasanton, CA, USA) is the most widely used contact fundus imaging system for ROP telescreening. Prior studies have indicated that compared to traditional bedside binocular indirect ophthalmoscopy (BIO), RetCam imaging has a high sensitivity and specificity [4,5,6,7,8,9,10]. However, the use of this imaging system for ROP screening has not been widely adopted because of its high price and limited portability.

Smartphones and advances in technology for capturing images have been used to detect multiple ophthalmologic diseases such as diabetic retinopathy [11,12], glaucoma [13,14], and anterior segment disorders [15]. The advantages of smartphone-based imaging systems include their ease of use, wide availability, quick photo sharing, and relative cost-effectiveness. The use of smartphone-based devices for ROP screening has been recently reported [16,17]. However, smartphone-based ROP screening has been conducted only in pilot studies. No prospective head-to-head comparison of systemic parameters related to safety and pain scores between RetCam and smartphone-based devices for ROP screening has been performed. Therefore, we conducted this study to compare the efficacy and safety between smartphone-based fundus photography images and RetCam for ROP screening in a prospective cohort.

## 2. Materials and Methods

### 2.1. Patients

This was a single-center, prospective study. The study period was from February 2020 to September 2020. The study was approved by the Institutional Review Board of the Linkou Chang Gung Memorial Hospital, Taoyuan, Taiwan (No.: 201901378A3), and adhered to the tenets of the Declaration of Helsinki. All legal guardians or parents signed consent before participating in this study.

The current national screening criteria in Taiwan are based on the 2013 screening policy of the American Academy of Pediatrics Section on Ophthalmology, American Academy of Ophthalmology, American Association for Pediatric Ophthalmology and Strabismus, and American Association of Certified Orthoptists Premature newborns with a birth weight (BW) <1500 g, gestational age (GA) ≤32 weeks, or birth weight 1500–2000 g, but with an unstable clinical course were screened for ROP 4–6 weeks after birth [18]. The inclusion criteria of the study were patients who met the screening criteria whose legal guardians or parents agreed to participate in this study and provided informed consent.

If the patient’s family refused to participate in the study or the clinical status of the patient was not stable for undergoing the examinations, the patients were excluded from the study.

General information of the patients including sex, GA, BW, postmenstrual age (PMA) at examination, and ROP status was documented.

The primary outcome of our study was the agreement of the zone and stage of ROP and the presence or absence of plus disease between photographic assessments and BIO results. The secondary outcomes were safety profiles, pain scores, and complication rates between the two imaging modalities during ROP screening.

### 2.2. ROP Screening

Thirty to sixty minutes before the examination, the patients’ pupils were dilated with 2.5% phenylephrine and 0.5% tropicamide. All the patients were first examined with BIO (Heine Omega 500, Heine Optotechnik, Gilching, Germany) by a certified ophthalmologist, and the results were viewed as the ground truth related to the patients’ ROP status. The patients’ eyes were opened with a sterile lid speculum. The stage and zone of ROP and the presence or absence of plus disease were recorded.

Three days after the BIO exam, the patients received a fundus image recording by a well-trained study nurse with an assistant. Fundus images were obtained with two systems. The patient received wide-field contact fundus imaging (RetCam 3) first, according to a previously published technique [19]. Lid speculum and scleral indentation were used during the examination. The standard 6-image set consisting of the pupil and five retinal fields, with the optic disc central, temporal, nasal, superior and inferior, were taken. After the patient rested for at least one hour, we used a second imaging device with a portable hand-held assisted instrument (C3 Funduscam; Colpen Products Pvt. Ltd., Gujarat, India), which was not yet approved by Food and Drug Administration, mounted on an iPhone 6s (Apple Inc., Cupertino, CA, USA). A Volk Pan retinal 2.2 lens (Volk Optical, Mentor, OH, USA) was for the video capture of the fundus of the eye according to the company instruction manual. Based on our experience, the smartphone-based examination may require 10 to 20 times of practice to become familiar with the instrument. In addition to the central field, video in the peripheral field was also tried with the tilt of the imaging device. Scleral indentation was not performed during the examination due to the lack of a spare hand to rotate the eye with a scleral indenter. The use of video allowed individual frames to be extracted after acquisition for use as still images, meaning the practitioner only needed to obtain a good view of the fundus during the procedure and did not require an additional action to activate the shutter release to achieve the desired view. The study nurse then scanned through the video and selected useful frames for image grading.

Complications during the examination or reasons for halting the examination such as bradycardia, hypoxia, or apnea were documented. Vital signs such as blood pressure, pulse rate, respiratory rate, oxygen saturation, and neonatal infant pain scale (NIPS) [20], were recorded before, during, immediately after, 10 min after, and 30 min after screening.

### 2.3. Image Grading

All the photographs were graded by a researcher who had not participated in the patients’ clinical care. The images were rated and classified by three experienced ophthalmologists (J.-Y.L., E.Y.-C.K., and W.-C.W.). According to our study group’s previous published article, the interobserver differences was 86% [21]. The grader was masked to the identity of the patient and the BIO results. The grader assessed the photograph quality (excellent, acceptable, and not gradable) by using the following criteria: a photograph was considered excellent if it was in focus, the entire posterior pole was visualized, and the grader could easily identify the dilation or tortuosity of vessels. A photograph was considered acceptable if it was overexposed, underexposed, or out of focus, but adequate for determining the presence or absence of vessel dilation or tortuosity. An ungradable photograph was one where the image was out of focus or obscured by glare or motion artifacts.

For each image set, the zone and stage of ROP and presence or absence of plus disease were determined according to the 2005 International Committee for Classification of Retinopathy of Prematurity diagnostic classification [22]. The grader compared the results for the zone and stage of ROP and presence or absence of plus disease between the RetCam and smartphone-based images in agreement with the ground truth obtained by BIO.

### 2.4. Statistical Analysis

Baseline characteristics are presented as the mean and range for continuous variables and as proportions for categorical variables. The chi-square test or Fisher’s exact test was used to judge the zone and stage of ROP, the presence or absence of plus disease, and the complication rates. The Mann–Whitney U test was used to analyze the examination time between the study groups. Differences across five time periods in systemic parameters and pain scores were assessed using repeated measures analysis of variance (ANOVA). The agreement between image exams and BIO results was measured by Cohen’s kappa coefficient κ statistics. A value of 0 implied no agreement beyond chance, whereas a value of 1 corresponded to perfect agreement between BIO and the photographs [23]. All the analyses were performed using SAS software (version 9.4; SAS Institute Inc., Cary, NC, USA). *p* < 0.05 was considered statistically significant.

## 3. Results

### 3.1. Demographic Data

**Figure 1:**
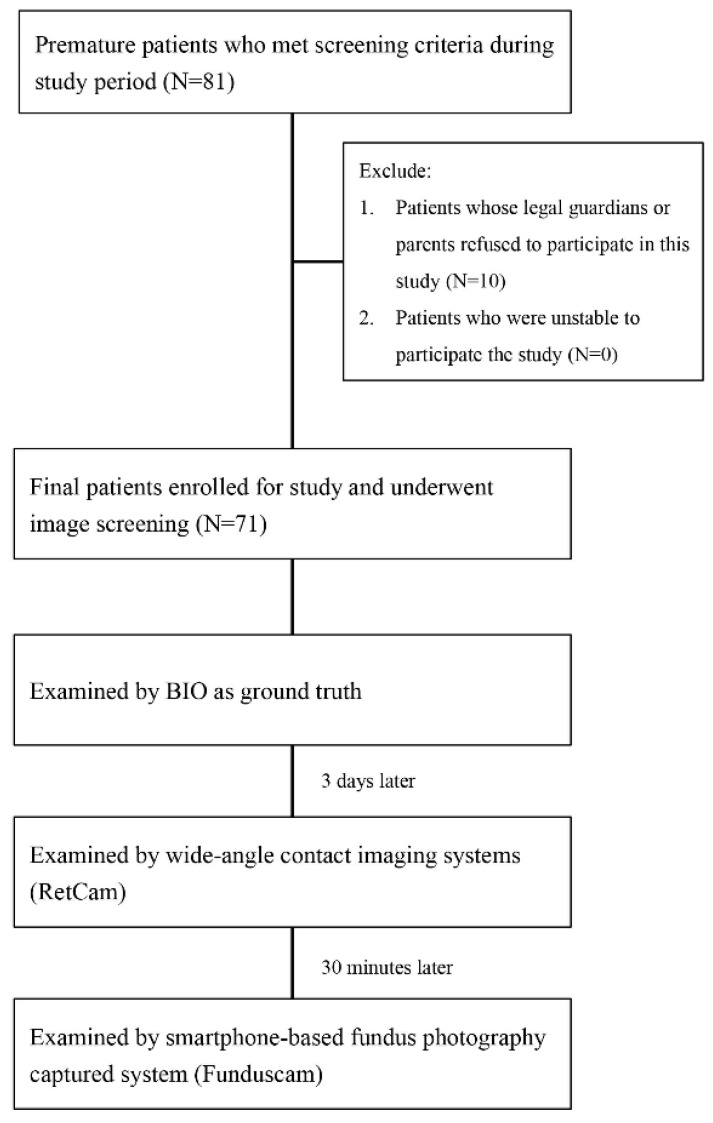
Flow chart and examinations performed in this study.

**Figure 2:**
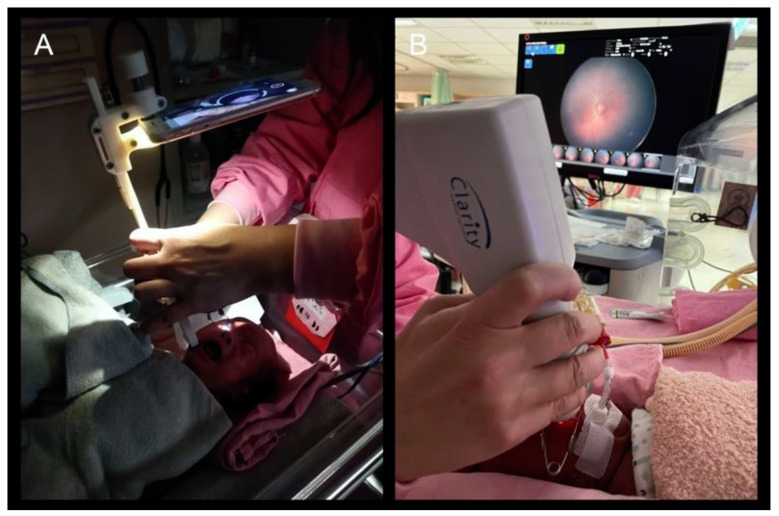
Image systems used in the study. (**A**) Smartphone-based fundus photography capture system (C3 Funduscam). (**B**) Wide-angle contact imaging system (RetCam).

The flowchart of recruitment is shown in Figure 1. A total of 71 premature babies (45 males and 26 females) were included in the study (Table 1). Typical photos captured with the C3 Funduscam and RetCam devices are shown in Figure 2A,B, respectively. Some patients received multiple examinations due to the need for continual follow-up. The total examination numbers were 198 eyes. The mean BW of all patients was 1055.8 g, and the mean GA was 28.5 weeks. The mean PMA when performing the examinations was 37.8 weeks. Of the 198 examined eyes, 85 (42.9%) were classified as having no ROP, 44 (22.2%) were classified as stage 1 ROP, 41 (20.7%) as stage 2, and 28 (14.1%) as stage 3. No eyes were classified as stages 4 or 5 ROP. For the zone determination, 14 eyes (7.1%) were classified as zone I, 98 (49.5%) as zone II, and 86 (42.9%) as zone III. Plus disease was noted in five eyes (2.5%), pre-plus disease in two eyes (1.0%), and no plus disease in 191 eyes (96.5%).

### 3.2. Image Quality and Ability to Judge ROP

The image quality of RetCam was assessed as not gradable in 0 eyes (0%), acceptable in 38 eyes (19.2%), and excellent in 160 eyes (80.8%) (Table 2). On the other hand, the image quality of C3 Funduscam was graded as not gradable in 17 eyes (8.6%), acceptable in 86 eyes (43.4%), and excellent in 95 eyes (48.0%). The image qualities between the two image modalities were significantly different (*p* < 0.001). The zone and stage of ROP could be judged in all the RetCam images. However, the grader could not judge the zone and stage of ROP in the C3 Funduscam images due to the limited view. Hence, the gradeability of the zone and stage of ROP between RetCam and C3 Funduscam was significantly different (both *p* < 0.001).

### 3.3. Agreement between Devices and Reference Standard

**Figure 3:**
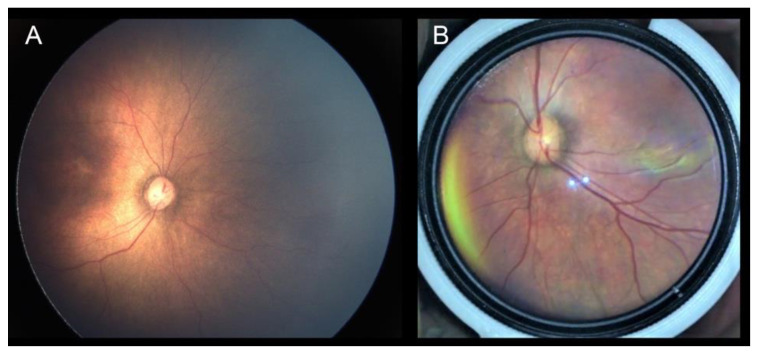
Examples of images taken by the different modalities. (**A**) RetCam. (**B**) C3 Funduscam.

The agreement between the imaging devices and the clinical examination by BIO is shown in Table 3. By using RetCam images (Figure 3A), the grader could perfectly classify the zone and stage of ROP and the presence or absence of plus disease. Cohen’s κ for plus disease, as a measure of agreement between RetCam and the ground truth exams, was 1.0. On the other hand, C3 Funduscam’s photos (Figure 3B) failed to reveal the zone and stage information, and the Cohen’s κ value for plus disease was 0.619.

### 3.4. Safety Profiles of Two Image Systems

The safety profiles of the two image modalities during different examinations are shown in Table 4. The results showed that regarding blood pressure, respiratory rate, and oxygen saturation, there were no significant differences between the two groups. However, heart rate showed a slight but significant difference between the two imaging systems (*p* for interaction <0.001). The NIPS scores, number of times an examination was halted, and ventilation adjustment times were similar between the two imaging devices (Table 5). The average time for image recording was significantly shorter for C3 Funduscam (5.0 ± 3.1 min) than for RetCam (7.7 ± 7.5 min, *p* < 0.001).

Regarding complications during examinations, 11 episodes were noted in the RetCam group, namely, seven episodes of apnea or hypoxia (7.1%), three of bradycardia (3%), and one of conjunctival hemorrhage (1.0%). In the C3 Funduscam group, apnea or hypoxia was noted four times (4%). There was no significant difference between the two imaging groups (*p* = 0.115).

## 4. Discussion

Our results showed that C3 Funduscam, a smartphone-based noncontact fundus photography device, could capture digital retinal photographs of prematurely born infants with adequate image quality but is unable to judge the zone and stage of ROP. The Cohen’s κ value for plus disease between BIO and C3 Funduscam was 0.619, indicating moderate agreement [23]. The safety profiles were good and showed no significant difference compared with those of RetCam. C3 Funduscam is not suitable for ROP screening because of the limited peripheral field visibility, which is vital for ROP severity discretion.

We found that the zone and stage of ROP could not be adequately judged from images captured by smartphone-based devices without scleral indentation. By using a Pan retinal 2.2 lens on the device, the field angle of C3 Funduscam was approximately 50 to 65 degrees. On the other hand, RetCam could provide a field angle up to 130 degrees. In addition, due to the noncontact designs of smartphone-based devices, it was difficult to capture high-quality video if patients were moving during the examination. Because the imager has to use both hands to hold the C3 Funduscam while filming the fundus, there is no spare hand to rotate the eye with a scleral indenter, so viewing the peripheral retina can be difficult.

Although it could not identify the zone and stage of ROP, C3 Funduscam showed fair capability in judging the presence or absence of plus disease, which requires information only from the posterior pole. In the Early Treatment of Retinopathy of Prematurity Randomized Trial (ETROP), the efficacy of early treatment for long-term favorable retinal structural outcomes in eyes with high-risk prethreshold or type 1 ROP was confirmed [24]. Additionally, plus disease is an important component in type 1 ROP, and timely intervention is needed to prevent its progression. The low cost makes cell phone fundus imaging systems available to resource-limited areas. The price of C3 Funduscam is USD 120, compared with RetCam, which is approximately USD 100,000. Additionally, the smartphone-based design makes the C3 Funduscam more portable and easier to store, and image files can be transmitted easily. Finally, documentation of ocular findings in premature infants is possible with this low-price device, although these images might not be able to show the whole area of the retina. Further improvement is needed to solve the critical issue of a limited angle field.

Wintergerst MWM et al. showed that by using smartphone-based fundus images, the sensitivity/specificity for the detection of plus disease and ROP was high (90%/100% and 88%/93%, respectively) [17]. The possible reasons that their results showed a higher detection rate for plus disease and for the zone and stage of ROP include the use of an additional light bulb and scleral indentation during image capture. However, they only examined 26 eyes of 14 patients, which was a relatively small cohort.

Examination of premature infants using BIO can trigger fluctuations in heart rate, blood pressure, and oxygen saturation. These changes may be due to a wide variety of causes including the oculocardiac reflex, systemic absorption mydriasis medications, scleral depression, application of the speculum to the eyelid, bright lights, and pain and stress caused to the infants [25,26,27,28,29,30]. The E-ROP study evaluated safety during ROP screening with RetCam [25]. It revealed sixty-five adverse events among 8311 total examinations with an adverse event rate of 0.8%. Importantly, no serious adverse events occurred in that study. Our study also showed good tolerability for patients using both the RetCam and C3 Funduscam systems. Vital signs such as blood pressure, respiratory rate, oxygen saturation, and pain scores, except for heart rate (*p* for interaction <0.001), were similar between the two image modalities (all *p* for interaction >0.05) and returned to baseline 10 min after the examinations. Heart rate was significantly slower when imaging with RetCam compared with C3 Funduscam (*p* for interaction <0.001). The possible reason for the difference in heart rate may be caused by the contact nature of RetCam, which induced an oculocardiac reflex and lowered heart rate [31]. The complication rates in both imaging systems were low, and no serious adverse events were observed.

Smartphone-based photography have several advantages including cost effectiveness, wide availability, and is easy to use [32]. It is also more portable and easier to store compared to the RetCam system. Additionally, smartphone technology has advanced significantly with better cameras, user friendly software, and faster processors in recent decades. Smartphone-based examination could be conducted without using a foot pedal to take pictures. The high accessibility makes smartphone-based screening for ophthalmic diseases a good choice for resource-poor communities and countries. Nevertheless, the biggest challenge of smartphone-based photography is to obtain detailed and wide-field images. A better optical designed product may help with solving these important problems. With the advanced technology in artificial intelligence, which may be applied to smartphone-based ROP screening [33], the efficacy and accuracy of smartphone-based photography may be improved in the future. Data sharing between smartphone-based photography and RetCam images could also be possible by using generative adversarial networks [34].

The limitations of our study include that it was a single-center investigation with a small number of enrolled patients. The number of patients with pre-plus and plus disease was limited. In addition, although we waited for at least one hour between the two different image screenings, it may still be possible that there were impacts on systemic parameters with previous examinations. Despite these limitations, our prospective study provided a fair comparison of the performance and systemic effects for imaging patients with ROP.

## 5. Conclusions

In conclusion, our study showed that the smartphone-based fundus imaging device C3 Funduscam had limited ability to view the peripheral retina and properly identify the zone and stage of ROP, thus making it unsuitable for clinical ROP screening practices without needing to worry about legal medical issues. The current model of this device needs further improvement before it can be reliably implemented in routine ROP screening.

## Figures and Tables

**Table 1:** Demographic data of the study population.

	Value
Patient numbers	71
Total examinations	99
Total examination eyes	198
Male, *n* (%)	45 (63.4)
GA (weeks), mean (range)	28.5 (23.6–35.3)
BW (grams), mean (range)	1055.8 (545.0–2000.0)
PMA at examinations (weeks), mean (range)	37.8 (30.4–57.0)
ROP stage (eyes), *n* (%)	
No ROP	85 (42.9)
Stage 1	44 (22.2)
Stage 2	41 (20.7)
Stage 3	28 (14.1)
Stage 4	0 (0)
Stage 5	0 (0)
ROP zone (eyes), *n* (%)	
Zone I	14 (7.1)
Zone II	98 (49.5)
Zone III	86 (42.9)
Plus disease (eyes), *n* (%)	
Plus disease	5 (2.5)
Pre-Plus	2 (1.0)
No plus	191 (96.5)

BW, birth weight; GA, gestational age; PMA, postmenstrual age at examination; ROP, retinopathy of prematurity.

**Table 2:** Image gradeability between the RetCam and smartphone-assisted devices (*n* = 198 in each modality).

	RetCam	Funduscam	*p*
	*n* = 198	*n* = 198	
Quality, *n* (%)			<0.001
Not gradable	0 (0)	17 (8.6)	
Acceptable	38 (19.2)	86 (43.4)	
Excellent	160 (80.8)	95 (48.0)	
Ability to judge zone, *n* (%)	198 (100)	0 (0)	<0.001
Ability to judge stage, *n* (%)	198 (100)	0 (0)	<0.001

**Table 3:** Agreement of image devices with clinical examination.

	Reference Standard (BIO)	Retcam	Funduscam	*p* ^1^	*p* ^2^
	*n* = 198	*n* = 198	*n* = 181		
Zone, *n* (%)				>0.999	<0.001
1	14 (7.1)	14 (7.1)	0 (0)		
2	98 (49.5)	98 (49.5)	0 (0)		
3	86 (43.4)	86 (42.4)	0 (0)		
Stage, *n* (%)				>0.999	<0.001
0	85 (42.9)	85 (42.9)	0 (0)		
1	44 (22.2)	44 (22.2)	0 (0)		
2	41 (20.7)	41 (20.7)	0 (0)		
3	28 (14.1)	28 (14.1)	0 (0)		
Plus, *n* (%)				>0.999	0.589
No plus	191 (96.5)	191 (96.5)	177 (97.8)		
Pre-plus	2 (1.0)	2 (1.0)	0 (0)		
Plus	5 (2.5)	5 (2.5)	4 (2.2)		
Plus disease agreement with BIO (Cohen’s κ value)	N/A	1.000	0.619	N/A	N/A

^1^ Comparison between BIO and Retcam; ^2^ Comparison between BIO and Funduscam; BIO, binocular indirect ophthalmoscopy; N/A, not available.

**Table 4:** Systemic parameters during different examinations.

	RetCam	Funduscam	*p* for Group	*p* for Time	*p* for Interaction (Group and Time)
	*n* = 99	*n* = 99			
**SBP (mmHg)**			0.011	<0.001	0.054
Before, mean ± SD range	78.1 ± 13.7 46–125	75.5 ± 12.8 51–108			
During, mean ± SD range	84.8 ± 15.6 43–126	86.3 ± 15.3 43–123			
Immediately after, mean ± SD range	86.3 ± 15.2 51–125	85.2 ± 14.5 46–125			
10 min after, mean ± SD range	81.3 ± 15.4 48–117	79.8 ± 14.5 50–117			
30 min after, mean ± SD range	81.5 ± 16.1 40–115	76.5 ± 12.5 52–111			
**DBP (mmHg)**			0.205	<0.001	0.571
Before, mean ± SD range	48.6 ± 14.2 23–99	46.6 ± 12.9 23–87			
During, mean ± SD range	54.9 ± 13.5 19–86	55.8 ± 15.6 24–96			
Immediately after, mean ± SD range	54.0 ± 14.9 16–97	53.3 ± 15.1 16–93			
10 min after, mean ± SD range	49.0 ± 14.6 19–89	48.6 ± 13.6 23–98			
30 min after, mean ± SD range	48.8 ± 14.6 20–97	46.2 ± 11.1 25–86			
**MBP (mmHg)**			0.041	<0.001	0.253
Before, mean ± SD range	58.4 ± 12.8 36–105	56.2 ± 12.0 37–94			
During, mean ± SD range	65.1 ± 12.7 30–94	66.1 ± 14.9 30–105			
Immediately after, mean ± SD range	65.2 ± 13.5 34–105	64.2 ± 13.5 29–104			
10 min after, mean ± SD range	60.2 ± 13.7 29–94	59.2 ± 13.0 32–103			
30 min after, mean ± SD range	60.1 ± 14.5 27–100	56.4 ± 10.6 36–91			
**HR (bpm)**			0.016	<0.001	<0.001
Before, mean ± SD range	159.2 ± 17.4 125–203	157.3 ± 15.5 122–201			
During, mean ± SD range	174.2 ± 30.5 78–225	189.8 ± 20.0 145–238			
Immediately after, mean ± SD range	181.4 ± 25.4 97–235	185.2 ± 20.8 126–224			
10 min after, mean ± SD range	163.0 ± 17.5 121–223	159.9 ± 16.3 126–210			
30 min after, mean ± SD range	155.9 ± 15.2 115–197	154.3 ± 14.1 126–197			
**RR (rpm)**			0.138	<0.001	0.495
Before, mean ± SD range	45.0 ± 13.7 22–90	45.6 ± 10.2 22–90			
During, mean ± SD range	51.5 ± 17.0 25–108	51.1 ± 15.3 25–112			
Immediately after, mean ± SD range	55.0 ± 17.2 30–105	54.2 ± 16.6 29–101			
10 min after, mean ± SD range	52.7 ± 12.5 27–85	50.3 ± 9.8 32–80			
30 min after, mean ± SD range	49.0 ± 14.2 21–93	46.4 ± 9.9 30–79			
**SpO2 (%)**			0.042	<0.001	0.114
Before, mean ± SD range	95.2 ± 5.1 78–100	96.7 ± 3.8 78–100			
During, mean ± SD range	94.6 ± 6.6 68–100	95.1 ± 6.1 67–100			
Immediately after, mean ± SD range	94.8 ± 5.5 78–100	94.3 ± 6.3 74–100			
10 min after, mean ± SD range	97.1 ± 3.5 86–100	97.0 ± 2.8 90–100			
30 min after, mean ± SD range	96.1 ± 4.4 79–100	97.4 ± 2.9 85–100			

bpm, beats per minute; DBP, diastolic blood pressure; HR, heart rate; MBP, mean blood pressure; rpm, respirations per minute; RR, respiratory rate; SBP, systolic blood pressure; SD, standard deviation; SpO2, oxygen saturation; mins, minutes.

**Table 5:** Pain score and adjustments during examinations.

	RetCam	Funduscam	*p* for Group	*p* for Time	*p* for Interaction (Group and Time)
	*n* = 99	*n* = 99			
NIPS			0.076	<0.001	0.063
Before, mean ± SD range	0.02 ± 0.2 0–2	0 ± 0 0–0			
During, mean ± SD range	7.0 ± 0.1 6–7	7.0 ± 0 7–7			
Immediately after, mean ± SD range	2.1 ± 1.5 0–7	2.5 ± 1.3 0–7			
10 min after, mean ± SD range	0.01 ± 0.1 0–1	0.01 ± 0.1 0–1			
30 min after, mean ± SD range	0 ± 0 0–0	0 ± 0 0–0			
Times of halting examination (times), *n* (%)			0.209		
0	88 (88.9)	95 (96.0)			
1	8 (8.1)	3 (3.0)			
2	3 (3.0)	1 (1.0)			
Ventilation adjustment (times), *n* (%)			0.498		
0	97 (98.0)	99 (100)			
1	2 (2.0)	0 (0)			
Examination time (mins), mean ± SD	7.0 ± 2.1	5.0 ± 3.1	<0.001		

NIPS, neonatal infant pain scale; SD, standard deviation.

## Data Availability

The data presented in this study are available on request. The data are not publicly available due to the data security policy of Chang Gung Memorial Hospital.
